# Role of microglia in HIV-1 infection

**DOI:** 10.1186/s12981-023-00511-5

**Published:** 2023-03-16

**Authors:** Ruojing Bai, Chengcheng Song, Shiyun Lv, Linlin Chang, Wei Hua, Wenjia Weng, Hao Wu, Lili Dai

**Affiliations:** 1 Center for Infectious Diseases, Beijing Youan Hospital, Capital Medical University, Beijing, 100069 China; 2 Department of Anesthesiology, Tianjin Medical University General Hospital, Tianjin, China; 3 Department of Dermatology, Beijing Youan Hospital, Capital Medical University, Beijing, 100069 China

**Keywords:** Microglial, ART, HIV-1, Neuroinflammation, HAND

## Abstract

The usage of antiretroviral treatment (ART) has considerably decreased the morbidity and mortality related to HIV-1 (human immunodeficiency virus type 1) infection. However, ART is ineffective in eradicating the virus from the persistent cell reservoirs (e.g., microglia), noticeably hindering the cure for HIV-1. Microglia participate in the progression of neuroinflammation, brain aging, and HIV-1-associated neurocognitive disorder (HAND). Some methods have currently been studied as fundamental strategies targeting microglia. The purpose of this study was to comprehend microglia biology and its functions in HIV-1 infection, as well as to look into potential therapeutic approaches targeting microglia.

## Introduction

Microglia are important for brain health because they engage in a variety of brain functions and help to maintain the physiological structure of the brain [1]. Microglia are cells in the brain’s innate immune system that may generate immune responses [2, 3], repair tissue damage, and contribute to the regeneration and reconstruction of the brain's structure and function. Microglia are known as central nervous system (CNS) macrophages, which are one of the primary cell reservoirs of hidden HIV-1 [7]. However, microglia may become uncontrolled or imbalanced in other situations, causing brain injury or pathological phenomena (e.g., neuroinflammation) and inducing or exacerbating neurodegenerative lesions [4, 5].

Even though effective antiretroviral therapy (ART) has significantly lowered human immunodeficiency virus type 1 (HIV-1) infection and mortality rates, no definitive HIV-1 treatment has been established. The major hurdle to HIV-1 eradication is the presence of latent HIV-1 in cells and anatomical reservoirs [6]. The cells mentioned above are considered to contribute to the occurrence of drug tolerance and reseed peripheral tissue. In addition, HIV-1-infected microglia induce neuroinflammation, accelerate brain aging, and promote HIV-1-associated neurocognitive disorder (HAND). Accordingly, eradicating the infected cells from the cerebrum is critical for achieving virus eradication. Therefore, learning more about microglia biology, their involvement in HIV-1 infection, and potential treatments will help prevent HAND and cure HIV-1.

## The biology of microglia

Microglia are CNS immunocytes discovered by outstanding scientists in the nineteenth and twentieth centuries [8]. At an early developmental stage in the yolk sac, microglia are produced from the monocyte/macrophage lineage rather than the neural lineage (e.g., neurons, astroglia, and oligodendroglia) [9, 10]. Microglia precursor cells penetrate the developing brain and help to maintain brain development, homeostasis, structure, and functions [11–14]. Numerous macrophage populations within and surrounding the brain should be addressed in addition to the parenchymal microglia in the brain tissue (e.g., meningeal, perivascular, and choroid plexus macrophages) [15].

In recent years, evidence has accumulated to support the hypothesis that microglia are immunoregulatory cells that constantly exert critical impacts on the CNS. They help to maintain normal cerebrum homeostasis while also generating potent reactions in the presence of inflammation stimuli, damage, hypoxic-ischemic status, and other detrimental status affecting cerebral normal functions. Stimulated microglial cells take on an amoeboid form and replicate and migrate to the site of injury to defend the CNS [16]. Nevertheless, over-activation and/or persistent stimulation of microglial cells with excessive inflammation mediators may result in neurotoxicity [17]. Thereby, microglia can contribute to neurocognitive degeneration by increasing pro-inflammation chemotactic factors, cell factors, and neurotoxins, which can affect stellate cells and nerve cells and induce neuron injury [18].

## Roles in HIV-1 infection

### Susceptibility to HIV-1

The presence of HIV-1 and simian immunodeficiency (SIV) virus infection increases the percentage of activated monocytes as well as monocyte/macrophage turnover in tissue [19]. According to Rappaport et al., migration of the CD14^+^CD16^+^monocytes subpopulation, which is highly susceptible to HIV-1 infection, through the BBB into the CNS is essential in the pathogenesis of HAND [20].

Not only does HIV-1 require the presence of CD4 to enter CNS cells, but it also requires the presence of chemokine receptor type 4 (CXCR4) or CC-chemokine receptor (CCR) 5 co-receptors and CCR3 receptors to generate valid infections. It is worth noting that CCR5 is strongly linked to viral invasion and the progression of neurological diseases. CCR3 and CCR5 expression is detected on the surface of microglial cells, making them more susceptible to HIV-1 infection [21].

HIV-1 has been shown to infiltrate macrophages and microglia through CD4 mediation. Microglia infection is thought to be produced by infected mononuclear cell transmigration, which appears early in infections. In recent years, a subgroup of infected mononuclear cells, HIV^+^, CD14^+^, and CD16^+^ mononuclear cells, has been discovered to prefer penetrating the blood–brain barrier (BBB) [22]. The cells described above express junction proteins [e.g., activated leukocyte cell adhesion molecule (ALCAM), junctional adhesion molecule-A (JAM-A), and CCR2] that help them penetrate the BBB. However, those infected mononuclear cells might infect microglia.

On the other hand, Microglia may ingest infected CD4^+^ T cells that migrate to the cerebrum [23]. Although not demonstrated, this later causal connection may be more likely to promote viral proliferation than free virus exposure [24]. Regardless of the infection mechanism, cerebral microglia appear to be susceptible to HIV-1 infection. Infection happens in microglia even with the high expression level of cell restrict factor SAMHD1 [Sterile alpha motif (SAM) domain and histidine–aspartate (HD) domain 1] [25], which is probably attributed to its phosphorylation by cyclin-dependent kinase 1 (CDK1), that occurs in cells cycling between G0 and G1 status [26].

Microglia have been discovered to be susceptible to HIV-1 infection both in vitro and in vivo [27]. Cosenza et al. and Churchill et al. identified HIV-1 proteins, DNA, and RNA in microglia from autopsy tissues from HIV-1 patients, though it should be noted that the patients in question died from severe HAND [28, 29]. According to a new study, microglial cells were subjected in patients with suppressed virus levels who died of causes unrelated to HIV-1 [30]. The study employed a cohort of 16 patients on ART with confirmed long-term HIV-1 control from the National Neuro AIDS Tissue Consortium (NTTC). The researchers employed the high-specificity technique to identify and measure DNA and RNA of HIV-1 at the cell level. As revealed from the outcomes, perivascular macrophages and microglial cells had HIV-1 DNA, other than stellate cells. When HIV-1 RNA was not detected in cerebrospinal fluid (CSF) or blood, the researchers observed HIV-1 RNA in the described cells in 6 of 16 individuals, showing that viruses might be produced in the CNS.

Besides, as previous research has shown, microglia are prone to infections in vitro. Certain in vitro models of infection-prone human microglial cells have been developed [31–35]. Moreover, a few latency models based on the aforementioned models have been created and have proven to be useful tools for investigating the infection process and molecule-level causal connections underlying the prevalence and treatment of latent HIV-1 in microglial cells [21, 36]. It has been proven that viruses were discovered in the CSF of subjects on effective ART with non-detectable plasmatic HIV-1, implying that HIV-1 may also be generated in the cerebrum [37, 38].

### Main HIV-1 reservoir in the brain

The following are the standards for cellular reservoirs: (i) the identification of integrated DNA of HIV-1 in the host genome of long-life cells, (ii) the identification of causal links allowing the viruses to persistently exist in cellular reservoirs, including the causal links capable of establishing and maintaining a hidden infection, and (iii) forming of duplication-competent particulates after the stimulation of reservoirs [39]. Additionally, two criteria for a real reservoir have been presented in microglia, such as the detection of integrated HIV-1 DNA within long-life cells and the identification of causal links allowing the viruses to remain in cellular reservoirs [40]. Furthermore, due to ethical and technological obstacles, it is not possible to investigate whether microglia are capable of producing replication-competent viruses in humans.

Meanwhile, using immunohistochemistry and the new, highly sensitive in situ hybridization methods RNAscope and DNAscope, researchers detected HIV-1 in cerebral macrophages and microglia rather than astrocytes [30]. Additionally, the infection is believed to be unproductive. As a result, they may not serve as real HIV-1 reservoirs [41]. In contrast, it has been revealed that macrophages and microglia are susceptible to HIV-1 infection and assist a valid infection [27]. As aforesaid, the valid infection in microglia within the CNS is correlated with HAND in humans and animals (e.g., the macaques) [7]. For this reason, macrophages and microglia can be considered true reservoirs in the cerebrum.

Microglia are generated in the yolk sac from erythromyeloid progenitors and penetrate the developing CNS during the embryogenetic process [42]. On that basis, they are the predominant resident cells in the cerebrum that can function as cerebral macrophages. Due to their long half-life of years, they have a stable population [43]. In contrast to macrophages, they are capable of cellular division, allowing HIV-1 to survive in the cerebrum [44]. Moreover, according to a recent study, microglia are extremely sensitive to the HIV-1 virus [45]. Generally, the microglia may form one of the primary HIV-1 reservoirs in the cerebrum.

### Promotion of neuroinflammation

Microglia are long-lived cells that induce immunological responses, allowing peripheral immunocytes to penetrate the CNS and maintaining CNS homeostasis [46]. They may be the critical cells to initiate and sustain positive feedback loops of persistent inflammatory events within the CNS.

When microglial cells are activated, they undergo functional, morphological, and phenotypic changes, and in vivo positron emission tomography (PET) image formation has established the role of microglia stimulation in the HIV-1 infection process [47, 48]. Once microglia are stimulated, genetic expression variations induce facilitated growth and variations in cell signaling (e.g., the production and release of pro-inflammation cell factors, chemotactic factors, and effector molecules) [49, 50]. The release of the above-mentioned molecules [e.g., matrix metalloproteinases (MMPs) and ROS) directly damages nerve cells, resulting in neuronal function loss and neurotoxic effects [51, 52], which eventually facilitates microglia stimulation. Furthermore, virally induced chemokine secretion [e.g., CCL2 (C–C motif chemokine ligand 2)] promotes the dissemination of chronic inflammatory events [53–56]. CCL2 stimulates microglial cells, regulates their migratory activities, and facilitates self-proliferative activities [57] while recruiting peripheral macrophages and T cells to the CNS. More immune-mediated damage, microglia stimulation, and the recruitment of immune cells are induced by these cells.

As revealed from recent findings, HIV-1 infection is capable of creating inflammation surroundings induced by virus proteins [transactivator of transcription (Tat) and gp (glycoprotein) 120] and pro-inflammation cell factors [tumor necrosis factor-alpha (TNF-α), interleukin (IL)-8, IL-6, and IL-1β] [58, 59]. The HIV-1 Tat protein can stimulate the NLR and upregulate caspase-1 and IL-1β levels in microglial cells, resulting in the production of IL-6 and TNF-α, and the intensification of pro-inflammatory response [60]. Microarray analyses were conducted by adopting cerebrum specimens from HIVE sufferers, HIV/noE, and HIV-controls. Significant microglial genes (e.g., immunity activation and functions, kinases, phosphatases, and pro-/anti-apoptosis and neurotrophy factors) were found to undergo remarkable changes during the process of HIV-1 infections, demonstrating that microglial functions are damaged and exhibit a proinflammation trend [50]. Nevertheless, the specific mechanisms of HIV-1-associated chronic neuroinflammation should be studied further because they may be influenced by specific infectious pathogens as well as the subsequent immune response.

### Acceleration of brain aging

Inflammatory microglia are likely to trigger or speed up cerebral aging via the interference with the physiology repairment and restoration process [61]. HIV-1, in particular, infects microglia, causing severe neuroinflammation and neuronal death if left untreated, culminating in brain structure and function loss [62–64].

First, after being infected with HIV-1, microglia's ability to fight infection, and also their ability to repair and regenerate, may be impaired [65]. Secondly, microglia may generate excessive neurotoxic factors such as oxygen free radicals (ROS) (e.g., arachidonic acid, quinolinic acid, and nitric oxide) [66]. It has been found that ROS is associated with neuronal cell death, neuroinflammation, and corresponding neurodegeneration, all of which accelerate brain aging [67–69]. For this reason, HIV-1 infection of the nervous system can accelerate brain aging by triggering neurotoxic immune responses in microglia. Thirdly, infection-related cytokines may become uncontrolled, and inflammatory cytokines will take over, causing pathological damage, inhibiting regeneration and repair, and interfering with brain physiological functions. The relevant destructive factors consist of IL-17A [70–74] and TNF-α [75]. Specifically, IL-17A is a marker of a key T helper cell population implicated in the pathogenesis of autoimmune and degenerative disorders. The role of IL-17A has been shown to be varied, as it not only contributes to pathogenic inflammation but also supports innate-like acute immune responses, and it is widely accepted that IL-17A causes diseases by activating glial cells. During inflammatory conditions, BBB endothelial cells express tumor necrosis factor superfamily (TNFSF) receptors and interact with TNFSF ligands in soluble form as well as on invading immune cells. TNFSF receptors and ligands are also found on CNS-invading effector immune cells as well as CNS-resident cells. Hence, this receptor-ligand interaction has a significant influence on the outcome of neuroinflammatory disease. Inflammatory cytokines may be generated locally in the brain, while HIV-1 infection may cause peripheral inflammatory cytokine to be overproduced, i.e., cytokine storm, and released into the bloodstream, affecting the BBB and the entire brain [76]. Fourthly, HIV-1 may remain in the brain for a period of time, resulting in a persistent low-grade inflammatory response. Taber et al. discovered that HIV-1 infection increased microglial activation in the hippocampus and neocortex through an analysis of autopsy reports from chronically HIV-1-infected patients, demonstrating that HIV-1-associated chronic neuroinflammatory responses may result in decreased neurons as well as neuronal cell death [77]. On the other hand, chronic HIV-1 infection causes neuroinflammation as well as microglial activation [78]. There is also mounting evidence supporting the production and deposition of β-amyloid-like peptides, which are comparable to those found in Alzheimer’s disease [79, 80]. All of the conditions mentioned above can have a long-term impact on microglial function, resulting in chronic low-grade inflammation or dysfunctional microglia, which reduces brain regeneration and remodeling.

Microglia are also affected by the elevated systemic inflammatory immunological state, which results in decreased physiological neuroregeneration and remodeling [81, 82]. Inflammation is undoubtedly enhanced and exacerbated by recurring or chronic HIV-1 infections [83]. An elevated and persistent inflammatory state in the brain may cause neurodegeneration owing to enhanced neuronal cell death and reduced neurogenesis, impaired remodeling, and permanent neural network injuries, thus worsening or hastening brain aging [61]. Moreover, since the information on the cellular and molecular pathways through which microglia accelerate brain aging is limited, further study is needed in the future.

### Contribution to HAND

Microglia, one of the resident members of the mononuclear phagocytic family in the CNS, are evenly distributed in the CNS parenchyma and are crucial in maintaining the homeostasis and health of the CNS, anti-inflammatory and resisting pathogen invasion. Hence, one of the pathways leading to HAND is the reduction of microglia caused by HIV-1 infection and injury [84]. In addition, viral proteins, cytokines, and chemokines produced after microglia infection are the main factors that indirectly cause neuronal apoptosis. However, the etiopathogenesis of HAND remains elusive, whereas it is attributed to multiple factors (i.e., ART neurotoxic effects, HIV-1 duplication within the CNS from infected cellular reservoirs, CNS inflammatory events, Ca aberrant regulation, mitochondrion function disorder, drug abuse, as well as autophagy) [78].

HIV-1-related proteins include the structural protein gp120, as well as regulatory proteins such as Tat, viral protein R (Vpr), and negative regulatory factor (Nef). gp120 mainly comes from the secretion of infected microglia and virion shedding and can induce the production of TNF-α, IL-1β, IL-6, macrophage colony-stimulating factor (GM-CSF), ROS, etc., thus directly or indirectly causing neuronal apoptosis [61]. As a regulatory protein of HIV-1, Tat can promote the replication initiation and elongation of HIV-1 DNA. Meanwhile, Tat, as a transactivator, can control gene expression in infected and non-infected cells. In HAND, Tat has cytotoxic and pro-inflammatory effects, and HIV-1 Tat protein can enhance microglial K^+^ efflux, Ca^2+^ influx, upregulate cytokine and chemokine levels, etc.[60] Vpr has been demonstrated to induce neuronal apoptosis, cell cycle arrest, transcriptional activation of viral promoters, nuclear translocation of pre-integrated complexes, and apoptosis in infected cells during the G2 phase [85].

Besides, microglia can express HIV-1 co-receptors such as CXCR4 and CCR5, both of which are members of the G protein-coupled receptor family, and complete signal transduction via the G protein signaling system. After the receptors are activated by HIV-1 or related proteins, a large amount of intracellular Ca^2+^ influx induces the formation of intracellular free radicals, damages cells, and causes apoptosis. Activated microglia can also secrete pro-inflammatory factors, including TNF-α, IL-1β, monocyte chemoattractant protein-1 (MCP-1), chemokines, and NO [86]. These inflammatory substances can up-regulate p38 MAP kinase, phosphorylate apoptosis-related transcription factors, and induce microglial apoptosis [87]. Additionally, positive feedback can activate more microglia and release a series of neurotoxic substances and more cytokines, resulting in a wider range of neuronal damage.

Furthermore, microglia can release excitatory neurotoxins such as quinolinic acid, glutamate, L-cysteine, and arachidonic acid. Excessive glutamate causes over-activation of glutamate receptors on nerve cell membranes, mainly N-methyl D-aspartate (NMDA), Ca^2+^ influx, the release of oxidative stress substances and toxic lipids such as 4-hydroxynonenal and ceramides, and activation of intracellular apoptotic pathways, thereby causing neuronal apoptosis [88]. The release of neurotoxins can stimulate astrocytes and microglia to release excitatory amino acids, resulting in positive feedback.

Although most HIV-1 infected individuals are primarily asymptomatic, the virus can co-exist with immunity activation of the CNS/cerebrospinal fluid [89–91]. Latent or active HIV-1 can cause neurocognitive impairment. HAND is classified into three categories according to the degree of the dysfunction: 1) asymptomatic neurocognitive impairment (ANI), 2) mild neurocognitive impairment (MND), and 3) HIV-associated dementia (HAD). HAD is the most severe variant of HAND, characterized by severe dementia shown by a lack of concentration, apparent motor faults, and unstable behavior changes [88]. HAND remains an unresolved multifactor aggravating HIV-1 disease. According to recent research, except for inhibiting HIV-1 duplication within the CNS, no clinical trials of HAND treatment might be effective [92, 93]. Nevertheless, certain studies reported a decrease in cognition damage as impacted by ART, i.e., sufferers with a regulated virus load who discontinued antiretroviral therapies have reported improved cognitive functions and mitigated neuronal damage [93, 94].

In summary, excessive activation and/or persistent stimulation of microglial cells, as well as overproduction of inflammatory mediators, may result in neurotoxicity [17]. Microglia are capable of generating neurocognitive degeneration (e.g., different forms of HAND), thereby increasing pro-inflammation chemotactic factors and cell factors along with neurotoxins, which adversely influence stellate cells and nerve cells and induce neural injury [18].

## Potential therapeutic strategies

**Fig. 1:**
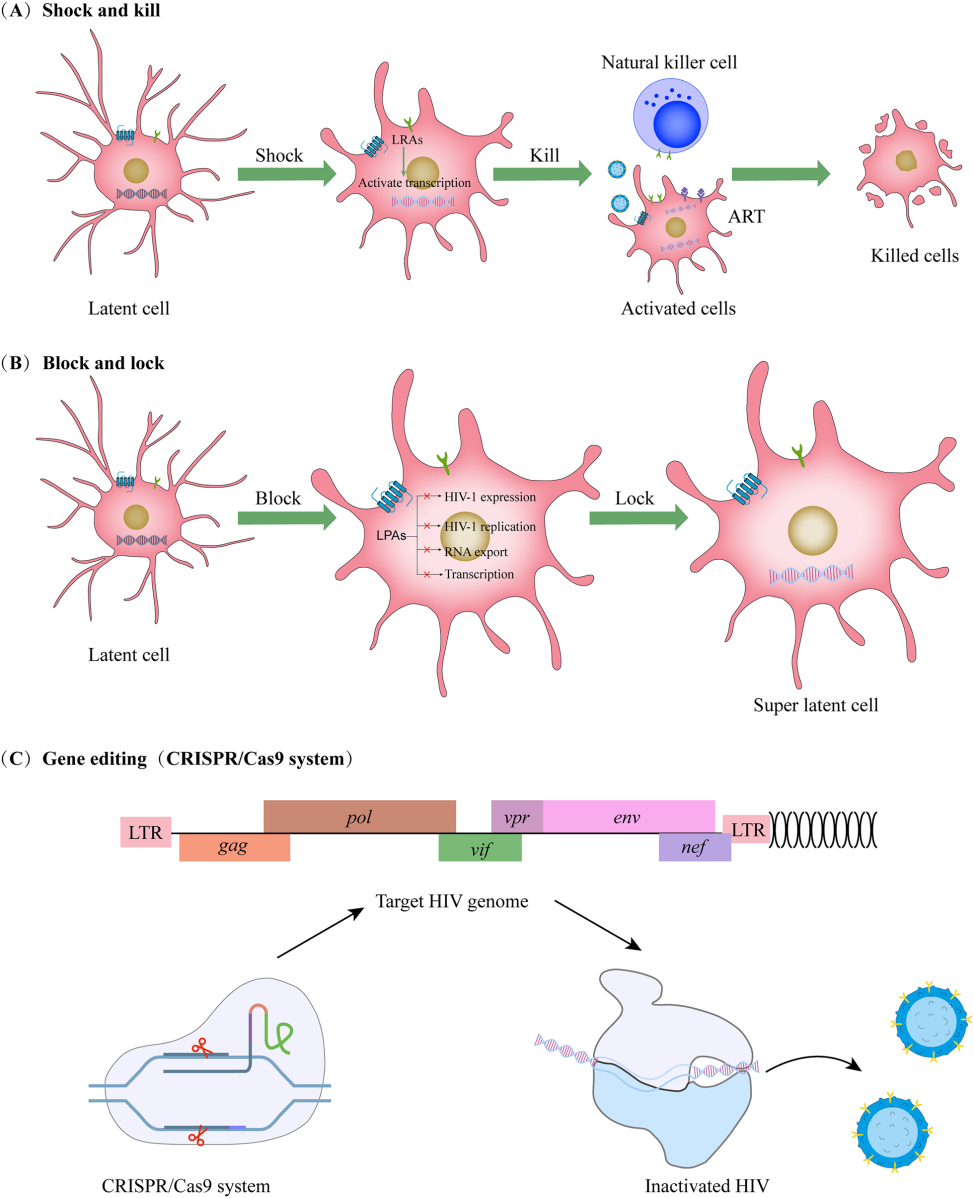
(A) Latency reversal agents were applied for the activation of HIV replication in latent cells. In this way, the immune system can target them targets for clearance. With virus expression reactivated, the strategy can remove reservoirs while targeting latently-infected cells. For the reactivation of transcription, latency reversing agents (LRAs) were explored. (B) Block and lock applied latency-promoting agents (LPAs) to block the transcription of HIV-1, thus inhibiting the processes of viral expression, such as replication, the transcription by Tat inhibition and RNA export. (C) Gene Editing (CRISPR/Cas9 system) draws upon a guided RNA and a Cas9 nuclease to remove targeted DNA sequences of cytokines while blocking the self-replication of virus

To eradicate the virus and provide a functional cure, it is critical to target the HIV-1 microglia reservoir in the CNS. As underlying measures for HIV-1, some strategies have been developed (Fig. 1).

### ART

Antiretroviral medications remain the most effective strategy to treat early-stage HIV-1 infection. The patients in the ANRS VISCONTI Study with regulated HIV-1 were able to manage HIV-1 duplication on their own once ART was stopped [95]. This condition was also discovered in "the Mississippi baby," an infant who got ART 30 h after being born. Previous research on early ART treatment in specific cohorts also provided data to support such a therapeutic protocol [96]. However, the virus did rebound in the Mississippi infant, indicating that early ART may be inadequate. Ongoing research by Sacha et al. is assessing the bone marrow reservoirs immediately posterior to infection, as well as the potency of early mega ART therapies in patients with AIDS in the early acute phases[97].

When treatment was started early, there were decreased levels of microglia stimulation and neuron injury biomarkers in the CSF [98]. An antiretroviral medicine with optimal entry into the cerebrum and minimal neurotoxic effects ought to be an evident option for virus inhibition. Unfortunately, since the majority of antiviral drugs are taken orally, their bioavailability in the CNS is limited, and absorption is slow due to the presence of the BBB. As a result, numerous drug delivery systems are now being evaluated in order to ensure that medications can penetrate the BBB, including invasive approaches such as intracerebral injections and implants, as well as BBB regulation employing supersound and penetration effects. Endogenetic transporters, pro-drugs, liposomes, nanoparticles, nano gels, dendrimers, and monoclonal anti-substances are examples of non-invasive techniques to deliver medications into the CNS [99]. Antiretroviral nanoparticle formulation is proposed as the best technique for improving BBB penetrance and site targeting. The U.S. FDA has approved several antiretrovirals that target the cerebrum across the BBB via an unexplained causal relationship. Some employ transportation proteins [e.g., P-gp, multidrug resistance-associated protein (MRP), and breast cancer resistance protein (BCRP)] [100]. ART does not adequately inhibit circulating virus-infected monocytes or macrophages, and in-depth studies should still be conducted.

### Shock and kill

Several compounds were studied for their ability to reactivate latent HIV-1, and some molecules were successfully created as latency-reversing agents (LRAs). The primary method of latent reversal is “Shock and Kill”, in which the LRA “shocks” the cellular reservoirs into expressing virus antigens and then “kills” them by exposing the stimulated cells to HIV-1-specificity CTLs (Fig. 1A) [101]. LRAs were applied to the reactivation target to determine and describe the cytokines of latent HIV-1. Considerable LRAs are being applied ex vivo and in clinical studies. Nevertheless, the primary focus is on circulating CD4^+^ T cells rather than microglia [102, 103]. 160 compounds have been used as LRAs, belonging to two major families or a third family that includes uncommon drugs with unusual or uncertain causative relationships (e.g., disulfiram and ixazomib) [104]. The screening of novel LRAs remains a field of intensive research [105]. The main disadvantage of adopting such approaches is that they enhance the cytotoxic response, which damages uninfected cells. The “Shock and Kill” technique does not remove reservoirs (for example, microglia) since their reactivation may promote brain inflammation and worsen HAND. Thus far, lncRNAs and miRNAs have been the most extensively studied epigenesis modulators [106, 107]. However, since HIV-1 transcription is unpredictable, the Shock and Kill methods did not contribute to the reactivation of all microglia with latent infection [7].

### Block and lock

The “Block and Lock” is known as another method to disable the capability of HIV-1 reservoirs to re-activate (Fig. 1B). LPAs are capable of suppressing HIV-1 transcription by triggering a deep latent state. They inhibit HIV-1 genetic expressions by triggering a profound latent status (the block), thereby avoiding HIV-1 genetic transcription (the lock) [108]. The first LPA found in a marine sponge was didehydro-cortistatin A (dCA), a chemical derivative of corticostatin. When combined with antiretroviral medication and LRAs, such inhibition can effectively prevent virus reactivation [98]. In microglia-like and astrocyte lineage cells, dCA has been shown to penetrate the BBB [109]. Even though dCA has an excellent inhibitory effect on CD4^+^ T cells, its activities in the CNS are unclear [110]. However, if a similar activity is discovered within CNS cells, dCA will be a widely recognized CNS medicine capable of significantly decreasing Tat-mediated neurotoxic effects while suppressing latent reversal. According to recent research, levosimendan suppresses acute HIV-1 duplication and the reactivation of hidden HIV-1 pro-virus in primary CD4^+^ T cells [111]. The aforementioned is a potential latency-facilitating measure that has been approved by FDA. On the other hand, its efficacy and/or toxic effects on brain cells should be evaluated to determine the underlying impact of eliminating CNS sanctuaries. Another substance, ABX4641, i.e., a suppressor of Rev that participated in the RNA exportation, prevents HIV-1 duplication in vitro tests and in animal models, whereas its efficacy remains unclear in the CNS [112].

Contrary to the previously mentioned, “Block and Lock” treatment is a procedure with great convenience since it is correlated with a decreased incidence of brain inflammation than “Shock and Kill.” This approach is similar to the "Shock and Kill" method in that the aim is to induce transcription and/or RNA exportation to counteract the activities of pro-inflammation cell factors while preventing viral protein synthesis [113]. As a result, combining the two approaches can facilitate the reduction of microglia reservoirs, although it may cause deep latent status in sanctuaries that aren't re-activated by LRAs.

### Gene therapy

Genetic therapies are widely regarded as a viable method of treating HIV-1 (Fig. 1C). The clustered regularly interspaced short palindromic repeats (CRISPR) and CRISPR-associated (Cas) systems refer to RNA-guided sequence-specific antiviral immune systems found in prokaryotic cells. In prokaryotes, small RNA molecules direct Cas effector endonucleases to invade foreign genetic material in a sequence-dependent manner, culminating in endonuclease-mediated DNA cleavage upon target binding. Additionally, a rewired CRISPR/Cas9 system in eukaryotic cells might be used for selective and precise genome editing. Therefore, CRISPR/Cas has been applied to target human pathogenic viruses as a potentially innovative antiviral strategy.

Zhang et al*.* [114] displayed the use of an inactive Cas9 (dCas9)-synergistic activation mediator (dCas9-SAM) system to re-activate HIV-1 in CD4^+^ T cells and microglia lines. This technology may have the benefit of influencing localized cells without HIV-1 in a minimal way. Hu et al. used a Cas9/guide RNA technique to eradicate the HIV-1 genome and immunize specific cells to resist HIV-1 re-activation in microglia, pre-monocytes, and T lineage cells with latent infection, producing the most promising results [115].

Drug penetrability in the CNS, where microglia reside, is a challenge that genetic therapies, like pharmacological treatments, fail to resolve. Meanwhile, another major limitation of genetic editing has been discovered: HIV-1 can evade CRISPR/Cas9-mediated suppression. Therefore, further research is required before it can be used in clinical applications.

## Conclusions

Early ART treatment has led to a significant decrease in HIV-1-related mortality in recent years. However, complete eradication is improbable unless concealed viral sanctuaries are targeted [116]*.* Involving and/or eradicating the aforementioned virus sanctuaries in clinical practice remains a difficult challenge. Microglia are useful for hidden reservoirs because they are resistant to viral cytopathy and have a long lifespan [117]. Hence, they are capable of persistently disseminating HIV-1. In addition, microglia act as virus reservoirs with inferior ART penetrability. Furthermore, microglia inflammation might trigger or speed up cerebrum aging via the interface with the physiology restoration process. In particular, HIV-1 infection inducing low-level neural inflammation might facilitate cerebrum aging and HAND. Some studies underlined the necessity of reactivating hidden sanctuaries with the optimum ART, improving cytotoxicity responses, and promoting programmed cell death in infected cells. Therefore, it is critical to target microglia, which is a difficult task. Moreover, combining the aforementioned innovative approaches with well-designed ART medications may be able to develop viable regimens for HAND by targeting infected microglia.

## Data Availability

Not applicable.

## Abbreviations

ALCAM: Activated leukocyte cell adhesion molecule
ANI: Asymptomatic neurocognitive impairment
ART: Antiretroviral therapy
BBB: Blood–brain barrier
BCRP: Breast cancer resistance protein
Cas: CRISPR-associated (Cas)
CCL2: C–C motif chemokine ligand 2
CCR: CC-chemokine receptor
CD4: Cluster of differentiation 4
CDK1: Cyclin dependent kinase 1
CNS: Central nervous system
CRISPR: Clustered regularly interspaced short palindromic repeats
CSF: Cerebrospinal fluid
CTLs: Cytotoxic T-lymphocytes
CXCR4: Chemokine receptor type 4
dCA: Didehydro-cortistatin A
dCas9-SAM: Inactive Cas9 (dCas9)-synergistic activation mediator
FDA: Food and Drug Administration
GM-CSF: Macrophage colony-stimulating factor
gp: Glycoprotein
HAD: HIV-associated dementia
HD: Histidine–aspartate
HIV/noE: HIV + patients without encephalitis
HIV-1: Human immunodeficiency virus type 1
HIVE: HIV encephalitis
IL: Interleukin
JAM-A: Junctional adhesion molecule-A
lncRNA: Long non-coding ribonucleic acid (RNA)
LPAs: Latency promoting agents
LRA: Latency reversing agent
miRNA: Micro-RNA
MMP: Matrix metalloproteinase
MND: Mild neurocognitive impairment
MRP: Multidrug resistance-associated protein
Nef: Negative regulatory factor
NLRC5: Nucleotide oligomerization domain (NOD)-like receptor C5
NLRP3: Nucleotide-binding domain leucin-rich repeat and pyrin-containing receptor 3
NMDA: N-methyl D-aspartate
NTTC: National Neuro AIDS Tissue Consortium
PET: Positron emission tomography
ROS: Oxygen free radicals
SAM: Sterile alpha motif
SIV: Simian immunodeficiency
Tat: Transactivator of transcription
TNFSF: Tumor necrosis factor superfamily
TNF-α: Tumor necrosis factor alpha
Vpr: Viral protein R
